# Supplementary material for: Frequency-specific attentional mechanisms phasically modulate the influence of distractors on task performance
Source: PLoS Biol. 2026 Feb 23;24(2):e3003664. doi: 10.1371/journal.pbio.3003664 (PMC12928401; doi:10.1371/journal.pbio.3003664)
Supplement: Attachment — Submitted filename: Response to Reviewers.pdf [file pbio.3003664.s008.pdf]

Below we respond to each comment from the reviewers. Our responses are in italics. We have also included any additional or edited text and any additional or edited figures. Thank you to all of the reviewers for their helpful feedback, which has greatly improved our manuscript.

Reviewer #1:

This study sought to provide evidence for distinct phases of the theta rhythm that either promote focusing on a target or make us susceptible to co-occurring distractors. The authors instructed subjects to detect a low-contrast gray target at one of four possible locations, which could appear either alone or in combination with a high-contrast colored distractor. Notably, the possible locations of both the target and the distractor were either validly or invalidly cued, and there was a variable time delay between the presentation of the cue and the target/distractor. The task execution was accompanied by the recording of the subjects' brain activity using an electroencephalogram (EEG). The authors found that behavioral measures including hit rate, false alarms, and d' were sensitive to the presence of distractors and the cue validity regarding both the target and distractor. The authors also found that the phase of oscillatory EEG activity across several frequencies was related to the behavioral measures. The strongest relationship was found between the 7 Hz theta phase and both hit rate and d' in situations with and without distractor. When a distractor was present, the authors reported only a significant relationship between theta phase and false alarms. Moreover, alpha phase was also related to behavioral measures in trials with distractors, occurring at occipital electrodes contralateral to distractors and modulated here the amplitude of the distractor-evoked visual response. Together, the authors conclude that the present study provided support for a rhythmic theory of attention that phasically modulates the influence of distractors on task performance.

We thank the reviewer for their thoughtful comments. This is a good summary of our work; however, there is one small point that should be clarified. On trials with distractors, the present results link hit rates, false alarm rates, and d' to pre-stimulus theta phase, whereas on trials without distractors, the present results link only hit rates and d' with pre-stimulus theta phase (not false alarm rates).

1. **Reviewer #1:** Previous studies on object-based attention have showed both behavioral and electrophysiological evidence for sampling (e.g., Fiebelkorn et al. 2018, Helfrich et al., 2018). I wonder whether the authors could show evidence for behavior-related theta phase effects on target processing in the face of distractors. This would provide a solid functional basis that distractor susceptibility fluctuates over time.

In agreement with prior reports (e.g., Fiebelkorn et al. 2018, Helfrich et al., 2018), the present results demonstrate a significant relationship between theta phase and target detection (i.e., as measured with hit rate and d'). This relationship between theta phase and target detection is also present on trials with distractors. Extending these findings, the present results further demonstrate a significant relationship between theta phase and false alarms on trials with distractors, with false alarms specifically being higher during the theta phase associated with worse target detection (i.e., the theta phase associated with lower hit rates). While we agree with the reviewer that it would be helpful to also link theta phase to electrophysiogical markers of target processing (i.e., target-evoked responses)—with and without distractors—there are a couple of reasons why this is not possible: (1) low-contrast targets in the present experiment did not evoke clear visual responses, and (2) even if there were clear target-evoked visual responses, it's not possible to isolate target-evoked responses from distractor-evoked responses (on trials with distractors), due to volume conduction. To clarify this limitation of the EEG data, we have included a new supplemental figure that compares evoked responses to the low-contrast target with evoked

responses to the salient distractor (i.e., demonstrating the absence of a clear target-evoked response). This new figure, S1 Fig, is pasted below. Although we are unable to test for phasic modulation of target-evoked responses, the present results do demonstrate phasic modulation of distractor-evoked responses (see Fig 9). More specifically, phase-specific increases in false alarms (on distractor trials) and phase-specific changes in distractor-evoked responses, both support the conclusion that susceptibility to distractors fluctuates over time (i.e., as a function of oscillatory phase).

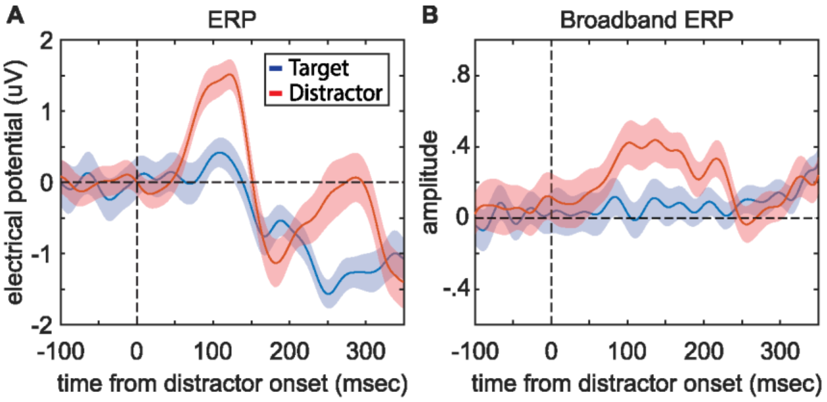

***S1 Fig. Target-evoked responses (i.e., associated with a low-contrast target) and distractor-evoked responses (i.e., associated with a salient distractor). A**, Grand average ERPs showing responses to targets without distractors (blue) and distractors without targets (red). **B**, As in **A**, but depicting the broadband (1-55 Hz) responses to the same stimuli. Error bars indicate ±1 SEM.*

2. **Reviewer #1:** The experiment involves manipulations of the presence and validity of target and distractor stimuli, resulting in nine conditions. The analysis of EEG data was conducted exclusively on validly cued targets that co-occurred with valid distractors or no distractors. However, invalidly cued targets and distractors were not analyzed due to low trial counts. Given that trial counts were already known prior to EEG data recording, did the authors a priori not intend to examine the effects of the validity manipulation? If so, I am curious about the rationale behind including these invalid target/distractor conditions in the experimental design, thereby reducing the trial counts for the manipulation of interest (distractor present versus absent). Alternatively, did the low trial count become apparent after recording due to excessive noise and inconclusive results? Was it not feasible to collect more EEG data, e.g., on separate days, in order to reach a sufficient trial count a priori? Please clarify.

It is common in attention-related studies to include a small percentage of invalidly cued trials for confirmation of attentional deployment. That is, better behavioral performance, e.g., on validly cued trials—relative to invalidly cued trials—confirms that participants were selectively deploying spatial attention based on spatially informative cues, as is confirmed in the present experiment (Fig 3). The number of trials in each condition was known in advance, and we never intended to look at phase-behavior relationships (or phase-ERP relationships) on invalidly cued trials. Our previous research on rhythmic sampling has similarly included invalidly cued trials for the sole purpose of confirming attentional deployment (e.g., Fiebelkorn et al., Neuron, 2018; Helfrich et al., Neuron, 2018). We acknowledge that the differences in phasic susceptibility to distractors between validly and invalidly cued trials is an interesting question for future studies; however, as the reviewer points out, this would require that participants complete multiple experimental sessions, given the necessarily low number of invalidly

cued trials. In response to the reviewer, we have clarified our motivation for including invalidly cued trials in the present experimental task:

From the Methods section (lines 499-503): "We included a small percentage of invalidly cued trials (i.e., 30%) to confirm that participants were selectively deploying spatial attention based on the spatially informative cues (Fig 3 and Fig 4). As with our previous work [14,16], the necessarily low percentage of invalid trials (i.e., low trial counts) precluded us from analyzing phase-behavior relationships associated with invalidly cued trials."

3. **Reviewer #1:** In order to find out whether rhythmic activity influenced target detection, the authors calculated a single value representing the strength of the phase-behavior relationship separately for electrodes, frequencies, behavioral measures and conditions (Fig. 6A-C). As the primary research questions concerned the impact of distractors on these phase-behavior relationships, I would be very grateful if the authors could directly compare the distractor and no-distractor conditions in this regard using cluster-based statistics? This approach, in my opinion, appears to be more appropriate than the one chosen here, where the comparison was limited to only five selected frequencies, one selected behavioral measure, and a global dissimilarity value that aggregated effects across electrodes. Also, there was no correction for multiple comparisons (Figure 6D).

We apologize to all of the reviewers that we failed to include a section describing our cluster-based approach to statistics, which we used for all of the phase-behavior analyses. This approach corrects for multiple comparisons (e.g., for the analyses shown in Fig. 6). We have added the following text to the manuscript:

From the Methods section (lines 610-628): "We used cluster-based permutation tests to determine the statistical significance of observed phase-behavior relationships, while correcting for multiple comparisons [86]. First, we used permutation tests to identify frequency x channel pairs with significant phase-behavior relationships. This was done by comparing each observed phase-behavior value (i.e., one-cycle sine fit)—averaged across subjects—with null values obtained by randomly shuffling phases across trials. After shuffling, we recalculated phase-behavior values for each subject and then averaged across subjects. These steps were repeated 1000 times to generate a null distribution. Phase-behavior relationships were considered significant for frequency x channel pairs that exceeded 95% of the null values. Next, we calculated the sum of phase-behavior values in clusters of significant frequency x channel pairs that were neighbors in either frequency or space (i.e., we calculated cluster sums). We then calculated the maximum sum of phase-behavior values in clusters obtained from the null datasets (i.e., the datasets obtained from randomly shuffling phases across trials). For each of the 1000 sets of null phase-behavior functions, we identified frequency x channel pairs with "significant" phase-behavior relationships. As described above for the observed data, null phase-behavior relationships that exceeded 95% of the remaining 999 null values for that frequency x channel pair were considered significant. Null cluster sums were then calculated across frequency and space, and the maximum cluster sum was kept (i.e., we kept the largest cluster sum from each iteration). This was repeated for the remaining 999 null datasets to generate a distribution of maximum cluster sums. Observed cluster sums that exceeded 95% of these null cluster sums were considered statistically significant."

Following the reviewer's suggestion, we now use a similar cluster-based approach to test for differences in phase-behavior relationships across the distractor and no-distractor conditions, separately for hit rates, false alarm rates, and d'.

From the methods section (lines 629-641): "We also adapted this cluster-based permutation approach to test for differences in the spectral and topographic characteristics of phase-behavior effects observed in

different conditions (distractor vs. no distractor). We calculated observed differences by subtracting the strength of the phase-behavior relationships (i.e., one-cycle sine fits) for the no-distractor condition from the corresponding values for the distractor condition. We then obtained null distributions by (i) randomly shuffling trials across the conditions (i.e., across the no-distractor and distractor conditions) for each subject, (ii) recalculating phase-behavior relationships for the shuffled conditions, (iii) averaging phase-behavior relationships across subjects, and (iv) recalculating the between-condition differences. Frequency x channel pairs with an observed absolute value (i.e., after subtracting strength of phase-behavior relationships for the no-distractor condition from strength of phase-behavior relationships for the distractor condition) greater than 95% of the absolute values of corresponding null pairs were considered significantly different. The calculation of cluster sums and the determination of statistically significant clusters proceeded as described above (i.e., for the analyses of within-condition phase-behavior relationships)."

The results of these additional analyses are now included in the Results section and Fig 6.

From the Results section (lines 260-273): "To further test how phase-behavior relationships differed between trials with and without distractors, we used (i) cluster-based permutation tests to compare phase-behavior relationships across trials with and without a distractor and (ii) global dissimilarity measures (DISS) to compare the phase-behavior topographies observed across these same conditions [50]. Cluster-based permutation tests revealed that while central theta effects (d') were apparent for both conditions, these effects were stronger for trials without distractors (Fig 6B-C). Trials with a distractor, in contrast, exhibited significantly greater coupling between alpha phase and all behavioral measures (HR, FAR, and d') at frontocentral electrodes and at posterior electrodes that were contralateral to the distractor (Fig 6B-C). Statistical comparisons of the topographies for phase-d' effects (distractor vs. no-distractor trials), from 6–10 Hz (Fig 7; $\alpha = .01$), revealed no differences at 6 Hz (DISS = 1.178; $p = .393$) and 7 Hz (DISS = 1.159; $p = .398$) and significant differences at 8 Hz (DISS = 1.497; $p = .008$) and 9 Hz (DISS = 1.485; $p = .009$). The test for 10 Hz (DISS = 1.469; $p = .015$) did not survive the correction for multiple comparisons. Combined, these analyses reveal (i) theta-dependent effects on trials with and without a distractor and (ii) alpha-dependent effects that only occurred on trials with a distractor."

We thank the reviewer for pointing out that we failed to correct our topographic analyses for multiple comparisons. We have edited the methods, results, and the associated figure (Fig 7) to reflect corrections for multiple comparisons in the topographic analyses (i.e., the global dissimilarity measures).

From the Methods section (line 652): "We used Bonferroni corrections to account for multiple comparisons ($\alpha = .01$)."

From the Results section (lines 267-271): "Statistical comparisons of the topographies for phase-d' effects (distractor vs. no-distractor trials), from 6–10 Hz (Fig 7; $\alpha = .01$), revealed no differences at 6 Hz (DISS = 1.178; $p = .393$) and 7 Hz (DISS = 1.159; $p = .398$) and significant differences at 8 Hz (DISS = 1.497; $p = .008$) and 9 Hz (DISS = 1.485; $p = .009$). The test for 10 Hz (DISS = 1.469; $p = .015$) did not survive the correction for multiple comparisons."

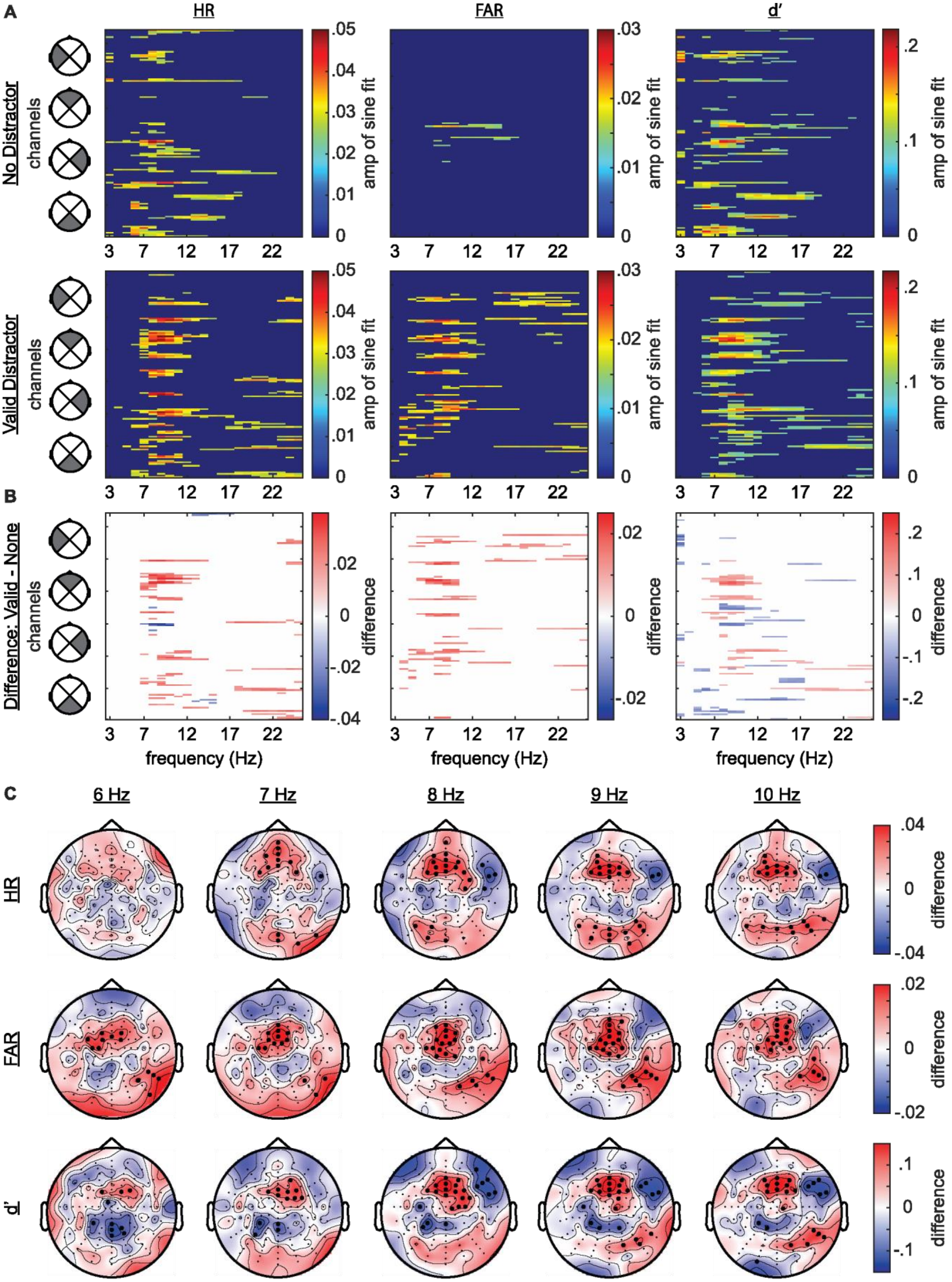
A
HR
FAR
d'
No Distractor
channels
amp of sine fit
Valid Distractor
B
Difference: Valid - None
difference
frequency (Hz)
C
6 Hz
7 Hz
8 Hz
9 Hz
10 Hz
HR
FAR
d'
difference

***Fig 6. Phase-behavior relationships. A**, Plots depicting the strength of statistically significant (p < .05) phase-behavior relationships as a function of frequency (3–25 Hz) across all 128 channels. Statistically insignificant results were set to zero. The first column depicts phase-HR relationships, the second depicts phase-FAR relationships, and the third depicts phase-d' relationships. The top row shows effects for trials with no distractor and the bottom row shows these effects for trials with a valid distractor. **B**, Plots depicting differences in phase-behavior coupling between conditions (valid distractor minus no distractor). Statistically insignificant results were set to zero. **C,** Topographies depicting the differences plotted in **B** for frequencies from 6-10 Hz (columns). The top row depicts phase-HR differences, the middle row depicts phase-FAR differences, and the bottom row depicts phase-d' differences. Channels with a significant effect at a given frequency are indicated by bold dots. The topographies are configured as if all stimuli appeared on the left side of the visual field (i.e., channels were flipped for trials with stimulus on the right).*

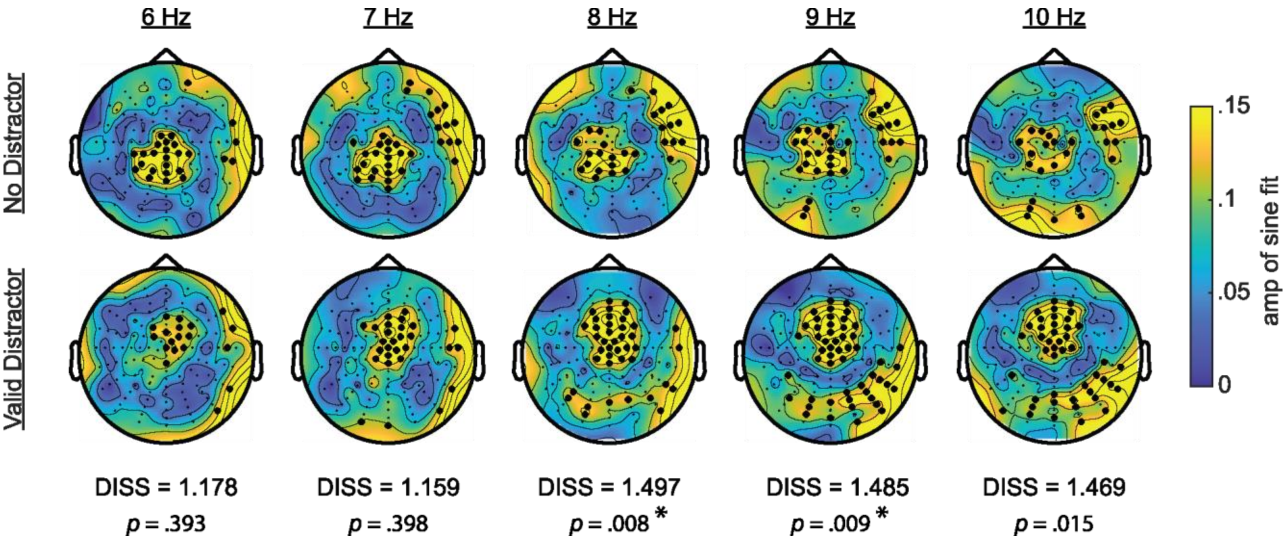

***Fig 7. Topographic analyses.** Scalp topographies show the strength of phase-d' relationships for frequencies from 6–10 Hz (columns). The top row shows effects for trials with no distractor and the bottom row shows effects for trials with a valid distractor. Channels with a significant effect at a given frequency are indicated by bold dots. Global dissimilarity measures (DISS) and p-values for tests comparing topographies across the no-distractor and valid-distractor conditions are listed below the respective frequency, with significant values after correcting for multiple comparisons (p < .05) indicated with an asterisk. The topographies are configured as if all stimuli appeared on the left side of the visual field (i.e., channels were flipped for trials with stimulus on the right).*

4. **Reviewer #1:** Figures 7C-D and 8C-D show results at electrode C23 at 7 Hz and C22 at 9Hz, respectively. I wonder whether the presented effects were limited to these single electrodes and frequencies? To obtain a more complete picture, I would appreciate if the authors could present results demonstrating the impact of the 'good' phase compared to the 'bad' phase across various frequency-behavioral measure combinations across all electrodes on distractor-evoked responses, after accounting for multiple comparisons.

Here, our frequency and electrode choices are constrained by previous findings, limiting the number of statistical tests. The frequencies are based on the present phase-behavior findings, with peaks at approximately 7 Hz and 9 Hz for significant phase-behavior effects (Fig 6 and Fig 7). Having isolated

oscillatory activity that phasically modulates behavioral performance, we next wanted to test for phasic modulation of distractor-evoked responses. This analysis was performed by taking the pre-stimulus phase on a trial-wise basis to group trials into 'good phase' and 'bad phase' bins (i.e., 'good' and 'bad' with respect to perceptual sensitivity). Distractor-evoked responses were then calculated separately for each of these bins. To get the trial-by-trial pre-stimulus phase for binning, we used the electrodes that demonstrated the strongest phase-behavior effects at 7 Hz (i.e., C23) and 9 Hz (i.e., C22). To select the electrode for calculating distractor-evoked responses, we a priori selected electrodes that have been repeatedly used in previous research on distractor-evoked responses (e.g., Gaspelin et al., 2023; Hickey et al., 2009; Redding & Fiebelkorn, 2024) and other studies of attentional effects on visual response components like the N2pc (e.g., Luck & Hillyard, 1994, Psychophysiology). That is, we used occipital channels A10/B7, which correspond to PO7 and PO8. In response to the reviewer, we have clarified the motivation for selecting these specific electrodes. We have also added a new supplemental figure (S4 Fig), pasted under our response to the next comment from Reviewer #1) that shows the topography of the difference wave for distractor-evoked responses during the "good" vs. "bad" alpha phases. This topography confirms that phasic modulation of the broadband response is occurring at the same electrodes as the distractor-evoked response (i.e., the distractor-evoked responses shown in Fig 4C), i.e., at electrodes repeatedly used in previous research on distractor-evoked responses (A10/B7).

From the Results section (lines 141-144): "All analyses of distractor-evoked responses used occipital electrodes A10/B7 (corresponding to PO7/PO8), which were selected a priori based on past work on distractor-evoked visual responses [42–44], as well as past work on other attention-related effects associated with visual responses, such as the N2pc component [45]."

5. **Reviewer #1:** Related to the points 3 and 4, if the objective was also to demonstrate that different frequencies (e.g., theta versus alpha) exhibit distinct contributions to the processing of distractors, I would appreciate if the authors conducted a direct statistical comparison of their effects, as contrasting a significant effect with a non-significant effect does not provide conclusive evidence regarding whether they differ significantly from one another.

In response to the reviewer, we have included a statistical comparison of phase-behavior effects on distractor-evoked responses across frequencies (7 and 9 Hz). This analysis is described in the methods and results sections and has been included as a supplemental figure (i.e., S4 Fig).

From the Methods section (lines 568-572): "We also compared ERP difference waves (good phase vs. bad phase) to test for differences in the effects of theta and alpha oscillations on distractor processing. For each of 1000 permutations, maximum cluster size was determined from a final difference wave created by randomly assigning trials to one of the two conditions (theta or alpha), separately for each subject. Observed clusters with a sum greater than 95% of null cluster sums were considered significant."

From the Results section (lines 285-288): "We also used a cluster-based permutation test to directly compare the influence of theta and alpha phase on distractor-evoked responses, revealing a significantly greater effect of alpha phase ($p = .024$) from 159.9 to 206.8 msec relative to distractor onset (S4 Fig). These results are consistent with an alpha-mediated gating of distractor processing [30]."

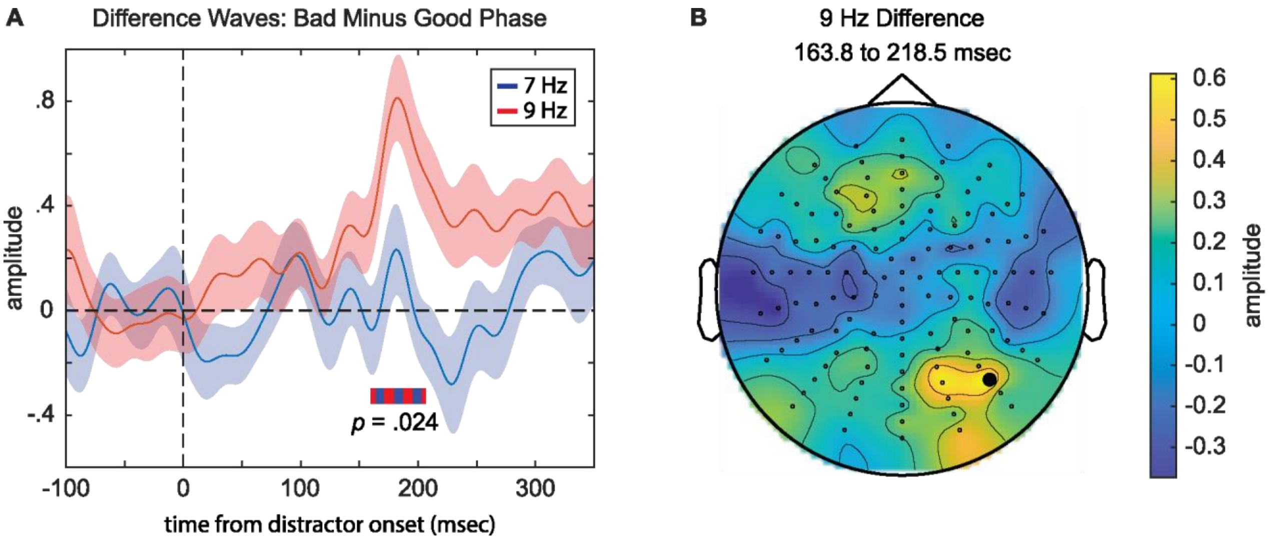

***S4 Fig. Comparison between phasic modulation of distractor-evoked responses at 7 Hz and 9 Hz. A**, Difference waves calculated from distractor-evoked broadband (1–55 Hz) responses as a function of pre-stimulus phase (‘good’ vs. ‘bad’) for 7 Hz (blue line) and 9 Hz (red line). Error bars indicate ±1 SEM. Horizontal blue and red bar indicates the time window of significant differences between conditions (159.9 to 206.8 msec). **B**, Topography of the difference wave for the 9 Hz effect averaged over the significant time window (163.8 to 218.5 msec). This plot is configured as if all stimuli appeared on the left side of the visual field (i.e., channels were flipped for trials with stimulus on the right). The electrode used for ERP analyses—A10 or B7 (corresponding to PO7 and PO8), depending on whether the distractor was presented to the left or right side of the visual field—is depicted with a bold dot.*

6. **Reviewer #1:** The experiments required subjects to focus on different locations. I'm curious to know how much the presented EEG results could be influenced by signals related to eye movements. Since the authors used an infrared eye-tracking camera to record eye movements, I would appreciate if they could address this concern.

By design, large saccades (> 2 dva) were excluded from the analyzed time periods. That is, we aborted trials when subjects broke fixation (by > 2 dva). Across subjects, microsaccades (< 2 dva) occurred on 7.51% of all trials (SD = 0.055). We specifically measured the occurrence of microsaccades during the window of the longest wavelet used for phase estimates in the present analyses (i.e., -666 to 0 msec relative to stimulus onset). Phase-behavior analyses were repeated after excluding trials with microsaccades, confirming the same general pattern of results. S3 Fig shows phase-behavior relationships for all trials relative to phase-behavior relationships for trials without microsaccades (i.e., after excluding trials with microsaccades).

From the Results section (lines 238-245): “Previous work has similarly linked fluctuations in the likelihood of microsaccades—fixational eye movement of < 2 degrees of visual angle (dva)—with frequency-specific phase [22]. As the direction of microsaccades are associated with attentional deployment and attentional shifts [48,49], we re-calculated phase-d’ relationships after excluding trials with microsaccades (7.5% of trials) that occurred prior to stimulus presentation (i.e., prior to the presentation of targets and/or distractors). As in previous work [14], this control analysis generally demonstrated the same pattern of phase-behavior relationships after the exclusion of trials with microsaccades (S3 Fig), confirming that phase-behavior relationships are not attributable to microsaccades.”

From the Methods section (lines 603-609): "Because previous work has also linked the likelihood of microsaccades with theta phase [22], we tested whether excluding trials with microsaccades affected the general pattern of phase-behavior relationships [14]. Saccades were detected using the EyeLink default speed threshold of 30°/s and acceleration threshold of 8000°/s^2. We specifically measured the occurrence of microsaccades (< 2 dva) during the window of the longest wavelet used for phase estimates in the present analyses (i.e., -666 to 0 msec relative to stimulus onset). Across subjects, microsaccades occurred on 7.51% of all trials (SD = 0.055)."

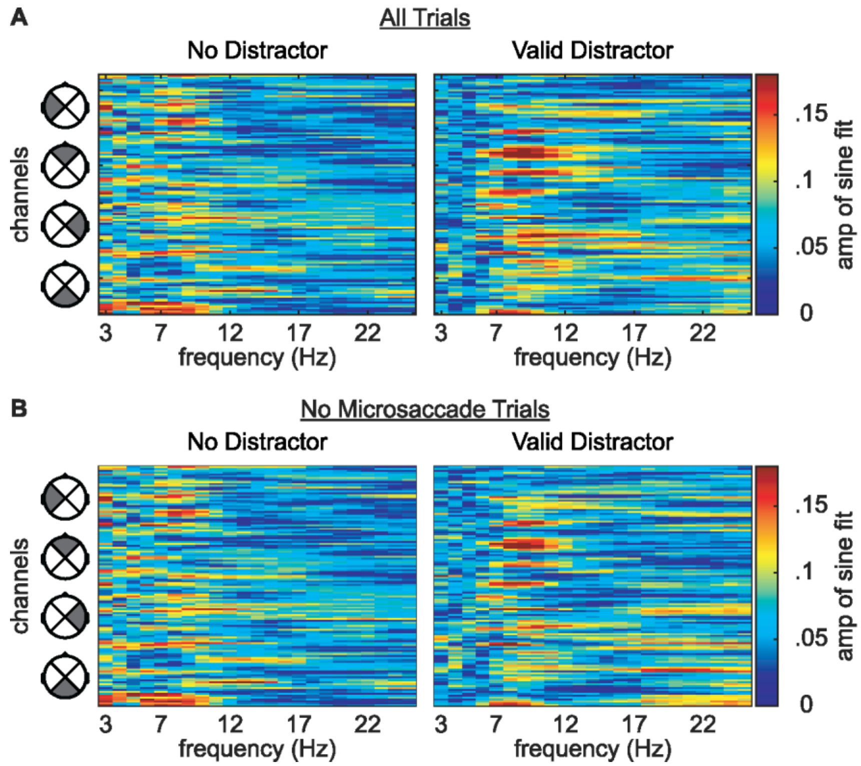

***S3 Fig. Phase-d' effects for all trials and trials without microsaccades. A**, Plots depicting the strength of phase-d' relationships as a function of frequency (3–25 Hz) across all 128 channels. **B**, As in **A**, but for only trials without microsaccades (< 2 dva).*

Reviewer #2:

This study investigates the distinct roles of theta and alpha rhythms in attention and distractor suppression, providing evidence for a core prediction of the Rhythmic Theory of Attention. The topic is timely and important. However, I have several major and minor concerns that should be addressed before the manuscript can be considered further.

Major Points
1. **Reviewer #2:** Potential luminance confound (line 425-426). The authors state that target luminance was adjusted during the task, but the details of this manipulation are not described. This raises a potential confound: comparisons between distractor and no-distractor conditions may also reflect systematic luminance differences. This could provide an alternative explanation for a key finding namely,

that the alpha phase-behavior effect occurs only on distractor-present trials. The authors interpret this as distractor-specific suppression, but it may instead reflect differences in target contrast (i.e., higher luminance). How can the authors rule out the possibility that the alpha effect is driven by luminance differences rather than distractor suppression?

We apologize for not including more details regarding our performance tracker and the related adjustments to target luminance. Those details have now been added to the manuscript (and pasted below). Importantly, although luminance was occasionally adjusted during the experiment, it was consistent across all of the interleaved trial types. That is, target luminance did not differ across the distractor and no-distractor trials.

From the Methods section (lines 479-484): "Target luminance was adjusted throughout the task using single integer adjustments to RGB values if hit rate (HR) fell below 50% or rose above 70% for a series of 10 valid-target trials. Target luminance was also adjusted up if false alarm rate (FAR) rose above 30% for a sequence of 10 trials with a false alarm or correct rejection at the cued target position. Due to random interleaving of trials, target luminance was independent of any experimental variables (e.g., distractor presence)."

2. **Reviewer #2:** Independence of theta and alpha effects (line 509-512).
The authors conclude that theta (~7 Hz) and alpha (~9-10 Hz) reflect functionally distinct oscillatory mechanisms. This interpretation relies on the two effects being spectrally independent. However, the time-frequency analysis employed may not provide sufficient resolution. The use of a 2-cycle Morlet wavelet for the 3-8 Hz range yields a bandwidth (e.g., ~3.5 Hz at 7 Hz) that overlaps substantially with the 9-10 Hz range. Thus, the apparent theta effect may be contaminated by adjacent alpha activity. To substantiate the claim of independence, the authors should apply methods with higher spectral resolution.

To measure phase as close to stimulus onset as possible, while also avoiding inclusion of stimulus-evoked activity, we needed to limit the width of lower-frequency wavelets. As indicated by the reviewer, this choice sacrifices spectral resolution. We now more clearly acknowledge this limitation.

From the Discussion section (lines 346-352): "It should be noted that we limited the width of the wavelets used to measure frequency-specific neural activity (i.e., we used a low number of cycles). This was done to obtain phase measurements that were as close as possible to stimulus onset (and subsequent behavioral measures), while also avoiding stimulus-evoked activity. There is therefore considerable spectral smearing across the theta- and alpha-frequency ranges. The present results, however, are consistent with distinct oscillatory mechanisms based on differences in peak frequency, topographical patterns, and functional relationships (e.g., alpha-specific links to distractor processing)."

Although effects are smeared across the theta and alpha ranges, different aspects of the phase-behavior topographies peak at different frequencies within the theta and alpha ranges. We demonstrate these differences by using topographic analyses (i.e., global dissimilarity measures) to compare trials with a distractor to trials without a distractor (Fig 7). To further test for such differences (as suggested by Reviewer #1), we now also use cluster-based permutation tests to compare the strength of phase-behavior effects across trials with distractors and trials without distractors (Fig 6B-C). These analyses further highlight effects that specifically emerge on distractor trials, peaking within the alpha-frequency range. For example, lateralized posterior effects (i.e., phase-behavior effects that occur contralateral to distractor locations).

From the Results section (lines 260-273): "To further test how phase-behavior relationships differed between trials with and without distractors, we used (i) cluster-based permutation tests to compare

phase-behavior relationships across trials with and without a distractor and (ii) global dissimilarity measures (DISS) to compare the phase-behavior topographies observed across these same conditions [50]. Cluster-based permutation tests revealed that while central theta effects (d') were apparent for both conditions, these effects were stronger for trials without distractors (Fig 6B-C). Trials with a distractor, in contrast, exhibited significantly greater coupling between alpha phase and all behavioral measures (HR, FAR, and d') at frontocentral electrodes and at posterior electrodes that were contralateral to the distractor (Fig 6B-C). Statistical comparisons of the topographies for phase-d' effects (distractor vs. no-distractor trials), from 6–10 Hz (Fig 7; α = .01), revealed no differences at 6 Hz (DISS = 1.178; p = .393) and 7 Hz (DISS = 1.159; p = .398) and significant differences at 8 Hz (DISS = 1.497; p = .008) and 9 Hz (DISS = 1.485; p = .009). The test for 10 Hz (DISS = 1.469; p = .015) did not survive the correction for multiple comparisons. Combined, these analyses reveal (i) theta-dependent effects on trials with and without a distractor and (ii) alpha-dependent effects that only occurred on trials with a distractor."

Further functional differences between 'theta effects' and 'alpha effects' emerge when evaluating whether there is phasic modulation of distractor-evoked visual responses. Whereas theta phases associated with better and worse behavioral performance showed no effect on the visual responses to salient distractors (Fig 8D), the alpha phase associated with worse behavioral performance was associated with stronger distractor responses, relative to the alpha phase associated with better behavioral performance (Fig 9D).

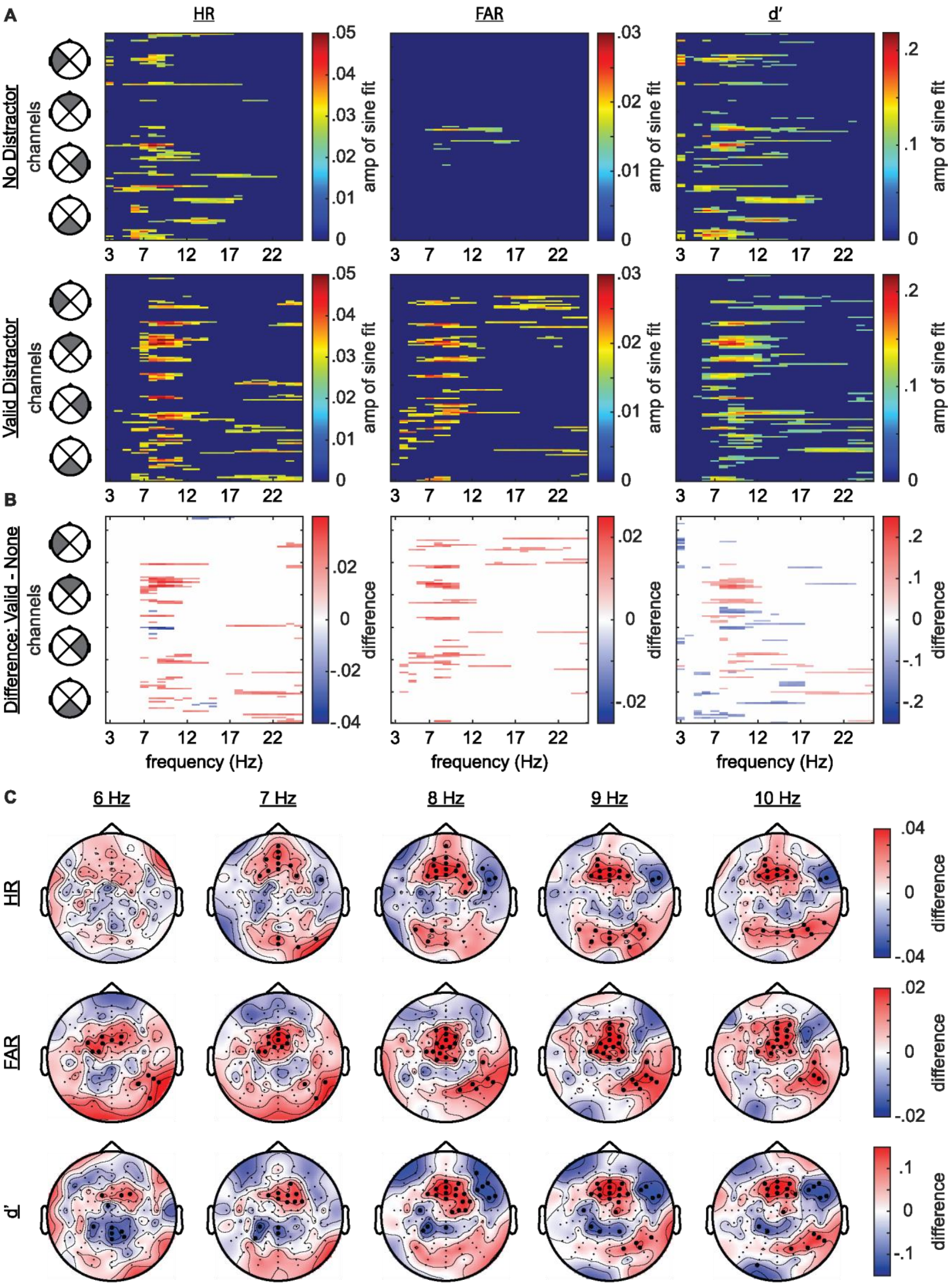

A
HR
FAR
d'
No Distractor
Valid Distractor
channels
amp of sine fit
3 7 12 17 22
B
Difference: Valid - None
difference
frequency (Hz)
C
6 Hz
7 Hz
8 Hz
9 Hz
10 Hz
HR
FAR
d'

__Fig 6. Phase-behavior relationships. A__, Plots depicting the strength of statistically significant (p < .05) phase-behavior relationships as a function of frequency (3–25 Hz) across all 128 channels. Statistically insignificant results were set to zero. The first column depicts phase-HR relationships, the second depicts phase-FAR relationships, and the third depicts phase-d' relationships. The top row shows effects for trials with no distractor and the bottom row shows these effects for trials with a valid distractor. __B__, Plots depicting differences in phase-behavior coupling between conditions (valid distractor minus no distractor). Statistically insignificant results were set to zero. __C,__ Topographies depicting the differences plotted in __B__ for frequencies from 6-10 Hz (columns). The top row depicts phase-HR differences, the middle row depicts phase-FAR differences, and the bottom row depicts phase-d' differences. Channels with a significant effect at a given frequency are indicated by bold dots. The topographies are configured as if all stimuli appeared on the left side of the visual field (i.e., channels were flipped for trials with stimulus on the right).

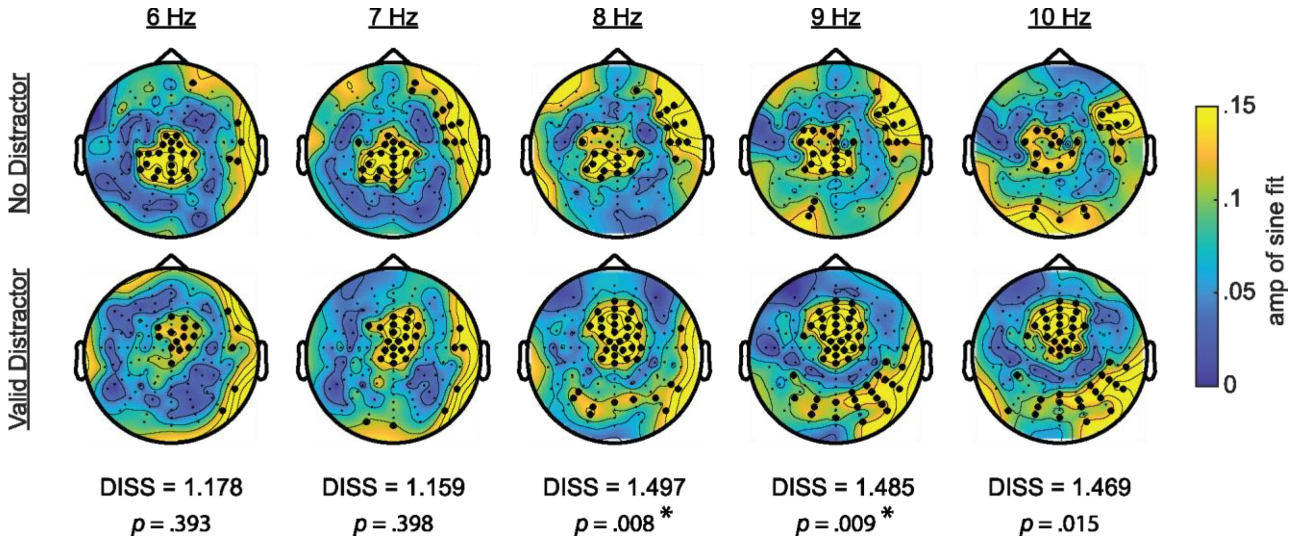

***Fig 7. Topographic analyses.** Scalp topographies show the strength of phase-d' relationships for frequencies from 6–10 Hz (columns). The top row shows effects for trials with no distractor and the bottom row shows effects for trials with a valid distractor. Channels with a significant effect at a given frequency are indicated by bold dots. Global dissimilarity measures (DISS) and p-values for tests comparing topographies across the no-distractor and valid-distractor conditions are listed below the respective frequency, with significant values after correcting for multiple comparisons (p < .05) indicated with an asterisk. The topographies are configured as if all stimuli appeared on the left side of the visual field (i.e., channels were flipped for trials with stimulus on the right).*

3. **Reviewer #2:** Cluster-based statistics (Fig. 6).
The significant phase-behavior relationships in Fig. 6 are reported to rely on cluster-based statistics, but the Methods section lacks sufficient detail. Specifically, the authors should clarify: The initial significance threshold (e.g., p-value) used to form candidate clusters. The dimensions along which clusters were defined (across adjacent channels, adjacent frequencies, or both). Providing these details is essential for reproducibility and proper evaluation of the reported effects.

We apologize to all of the reviewers that we failed to include a section describing our cluster-based approach to statistics, which we used for all of the phase-behavior analyses. We have now included this information in the methods section.

From the Methods section (lines 610-628): "We used cluster-based permutation tests to determine the statistical significance of observed phase-behavior relationships, while correcting for multiple comparisons [86]. First, we used permutation tests to identify frequency x channel pairs with significant phase-behavior relationships. This was done by comparing each observed phase-behavior value (i.e., one-cycle sine fit)—averaged across subjects—with null values obtained by randomly shuffling phases across trials. After shuffling, we recalculated phase-behavior values for each subject and then averaged across subjects. These steps were repeated 1000 times to generate a null distribution. Phase-behavior relationships were considered significant for frequency x channel pairs that exceeded 95% of the null values. Next, we calculated the sum of phase-behavior values in clusters of significant frequency x channel pairs that were neighbors in either frequency or space (i.e., we calculated cluster sums). We then calculated the maximum sum of phase-behavior values in clusters obtained from the null datasets (i.e., the datasets obtained from randomly shuffling phases across trials). For each of the 1000 sets of null phase-behavior functions, we identified frequency x channel pairs with "significant" phase-behavior relationships. As described above for the observed data, null phase-behavior relationships that exceeded 95% of the remaining 999 null values for that frequency x channel pair were considered significant. Null cluster sums were then calculated across frequency and space, and the maximum cluster sum was kept (i.e., we kept the largest cluster sum from each iteration). This was repeated for the remaining 999 null datasets to generate a distribution of maximum cluster sums. Observed cluster sums that exceeded 95% of these null cluster sums were considered statistically significant."

Minor Points

1） **Reviewer #2:** Sample size discrepancy. The alpha power analysis is reported as n = 27 after exclusions, but Fig. 9A (inset) is labeled n = 28. Please clarify and correct.

This should be n = 27. The figure (pasted below) has been amended to include the correct number.

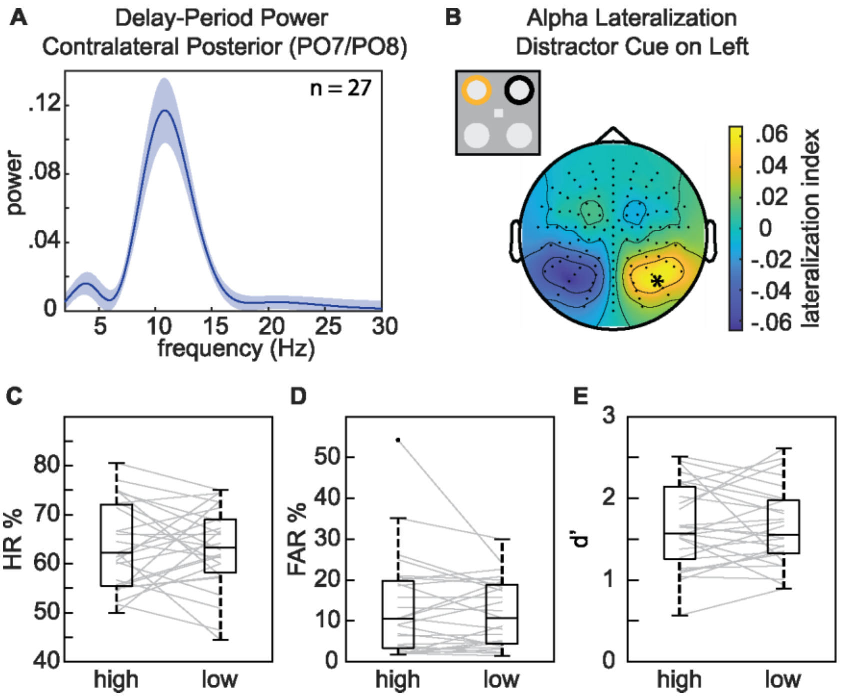

***Fig 10. Posterior alpha power modulation does not affect task performance. A**, Delay-period (-500 to 0*

msec relative to stimulus onset) power spectrum for frequencies from 3–30 Hz at occipital channels contralateral to the distractor cue. ***B****, Topography of alpha power lateralization relative to distractor cues presented on the left side. Channels were flipped across the midline for trials with the distractor cue on the right side. The asterisk indicates the channel used for binning trials by high and low alpha power lateralization.* ***C****, HR for trials binned by high and low alpha power lateralization using a median split. The line within each box represents the median, while the boundaries of each box represent the upper and lower quartiles, respectively. Lines outside the box represent maximum and minimum data points. Grey lines indicate the change across conditions for individual subjects.* ***D****, Same as* ***C*** *but for FAR.* ***E****, Same as* ***C*** *but for d'.*

2） **Reviewer #2:** Invalid condition trial counts. The phase-behavior analysis for invalid trials was omitted due to insufficient trial numbers (line 531-532). It would be helpful to report the average number of trials per phase bin in the invalid condition, to quantitatively support this exclusion.

It is common in attention-related studies to include a small percentage of invalidly cued trials for confirmation of attentional deployment. That is, better behavioral performance, e.g., on validly cued trials—relative to invalidly cued trials—confirms that participants were selectively deploying spatial attention based on spatially informative cues, as is confirmed in the present experiment (Fig 3). The number of trials in each condition was known in advance, and we never intended to look at phase-behavior relationships (or phase-ERP relationships) during invalidly cued trials. Our previous research on rhythmic sampling has similarly included invalidly cued trials for the sole purpose of confirming attentional deployment (e.g., Fiebelkorn et al., Neuron, 2018; Helfrich et al., Neuron, 2018). In response to the reviewer (and similar comments from another reviewer), we have clarified our motivation for including invalidly cued trials in the present experimental task:

From the Methods section (lines 499-505): "We included a small percentage of invalidly cued trials (i.e., 30%) to confirm that participants were selectively deploying spatial attention based on the spatially informative cues (Fig 3 and Fig 4). As with our previous work [14,16], the necessarily low percentage of invalid trials (i.e., low trial counts) precluded us from analyzing phase-behavior relationships associated with invalidly cued trials. In contrast to valid target/distractor conditions which included 180 trials prior to additional exclusions (e.g., minimum delay duration), each subject performed only 60 trials of each invalid condition."

3）**Reviewer #2:** Topographic comparison (Fig. 6D). The authors report p-values for differences between topographies. It is recommended to also report global dissimilarity, which would provide a more comprehensive metric of topographic differences.

We have now included global dissimilarity measures both in the results section and in Fig. 7.

From the Results section (lines 267-271): "Statistical comparisons of the topographies for phase-d' effects (distractor vs. no-distractor trials), from 6–10 Hz (Fig 7; α = .01), revealed no differences at 6 Hz (DISS = 1.178; p = .393) and 7 Hz (DISS = 1.159; p = .398) and significant differences at 8 Hz (DISS = 1.497; p = .008) and 9 Hz (DISS = 1.485; p = .009). The test for 10 Hz (DISS = 1.469; p = .015) did not survive the correction for multiple comparisons."

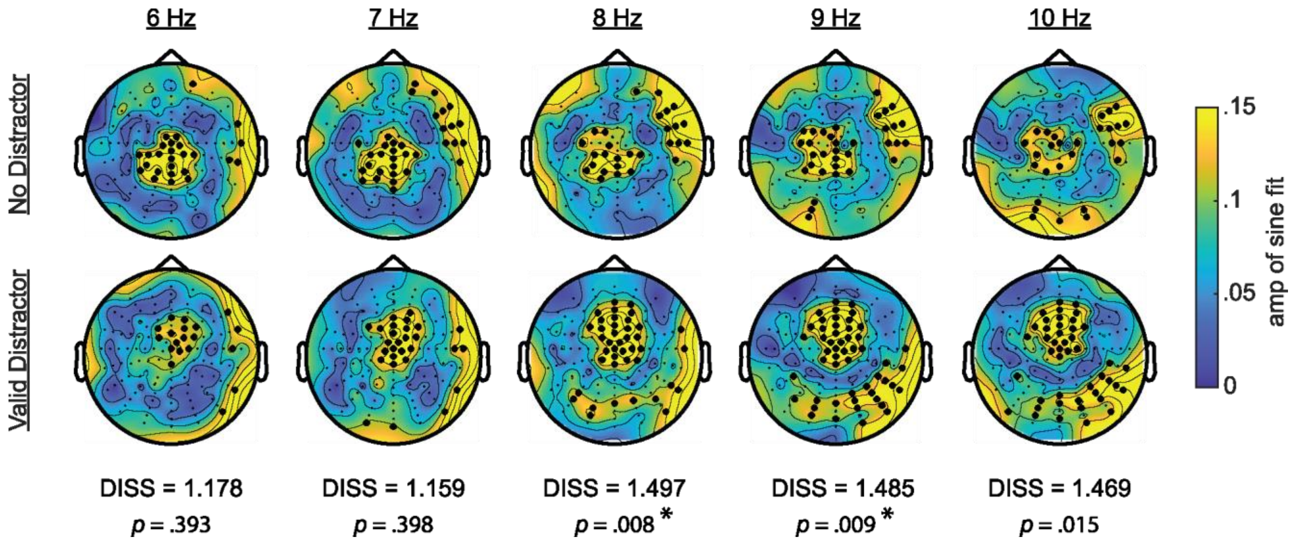

Fig 7. Topographic analyses. *Scalp topographies show the strength of phase-d' relationships for frequencies from 6–10 Hz (columns). The top row shows effects for trials with no distractor and the bottom row shows effects for trials with a valid distractor. Channels with a significant effect at a given frequency are indicated by bold dots. Global dissimilarity measures (DISS) and p-values for tests comparing topographies across the no-distractor and valid-distractor conditions are listed below the respective frequency, with significant values after correcting for multiple comparisons (p < .05) indicated with an asterisk. The topographies are configured as if all stimuli appeared on the left side of the visual field (i.e., channels were flipped for trials with stimulus on the right).*

4） **Reviewer #2:** Target-locked ERPs (lines 151-153). The manuscript notes that low-contrast targets did not elicit clear evoked responses, precluding tests of cue-related target enhancement. As this is a critical point, it would be useful to include the target-locked ERP waveforms in the Supplementary Materials.

We have added a supplemental figure (S1 Fig), illustrating the lack of an evoked visual response to targets presented without a distractor.

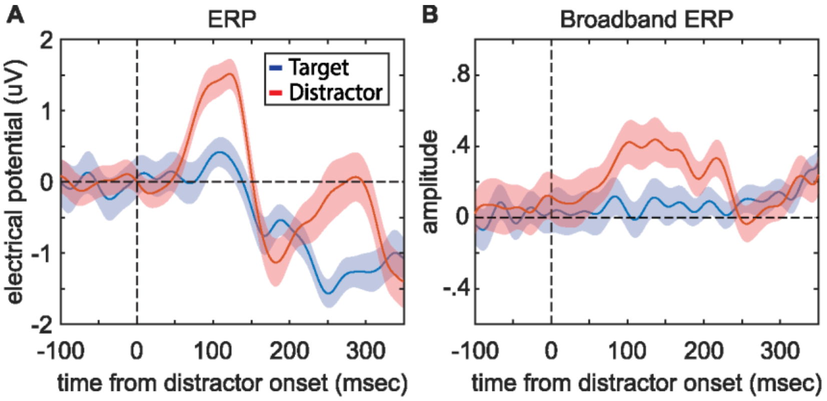

S1 Fig. Target-evoked responses (i.e., associated with a low-contrast target) and distractor-evoked responses (i.e., associated with a salient distractor). A*, Grand average ERPs showing responses to targets without distractors (blue) and distractors without targets (red).* ***B****, As in* ***A****, but depicting the broadband (1-55 Hz) responses to the same stimuli. Error bars indicate ±1 SEM.*

Reviewer #2: Overall, the study addresses an important question and provides potentially novel insights.

However, addressing the above methodological and interpretational concerns will be necessary to strengthen the validity and clarity of the conclusions.

We thank the reviewer for their thoughtful feedback, which has definitely improved the present manuscript.

Reviewer #3:

In this manuscript, Redding and collaborators are asking a timely question in the field of rhythmic attentional sampling, i.e., is the rhythmic sampling of the target co-occuring with a rhythmic sampling of the distractors thus generating rhythmic moments of facilitation of the distractors.

This is a very well designed and executed study. The paradigm is clever, and the behavior confirms the manipulation was successful, with the use of signal detection theory as a way of analyzing stimulus sensitivity. The authors carefully replicated previous empirical work before demonstrating theta-phasic effect on the distractors. The study is carefully contextualized and the literature well reviewed.

Together, the study is strong and brings compelling evidence in the current debate regarding the active processing of distractors.

Please add a dedicated section for code and data availability.

We thank this reviewer for their kind words regarding the project. We have included code and data availability information at the end of the manuscript.

From the Manuscript (lines 676-677): "All data files and code used for the main and supplementary analyses are available from The Open Science Framework at https://doi.org/10.17605/OSF.IO/V24GU."

Reviewer #4:

(Biao Han signed his report): This manuscript investigates how theta- and alpha-band oscillations modulate attention and distractor processing. The topic is timely and the experiments are well designed, but several core methodological aspects remain unclear. In particular, the analyses underlying the ERP, phase-behavior, and power results involve critical choices that are insufficiently justified or statistically unverified. Below I outline major methodological concerns that need to be addressed to ensure the robustness of the conclusions.

We thank the reviewer for their thoughtful comments, which have improved our manuscript.

1. **Reviewer #4**: ERP electrode selection and spatial multiple-comparison control
The authors state that "Occipital channels A10/B7 were used for all ERP analyses." The rationale for focusing on these specific electrodes requires clarification. Because the EEG was recorded with a 128-channel BioSemi cap, restricting analyses to one or two electrodes raises concerns about spatial bias and inflated Type I error. It should be explained whether A10/B7 were defined a priori, for example based on canonical occipital P1/N1 loci reported in prior work, or whether they were selected post hoc from the present dataset. The latter would constitute a circular analysis problem that may overestimate significance. Moreover, testing multiple channels and reporting a single significant site without correction virtually guarantees false positives in a 128-electrode montage. A more appropriate approach

would be to perform a cluster-based permutation test across occipital or all scalp electrodes to control for spatial multiple comparisons. If the choice of A10/B7 was indeed pre-specified, the authors should make this explicit and cite the supporting literature and topographical evidence.

To select the electrode for calculating distractor-evoked responses, we a priori selected electrodes that have been repeatedly used in previous research on distractor-evoked responses (e.g., Gaspelin et al., 2023; Hickey et al., 2009; Redding & Fiebelkorn, 2024) and other studies of attentional effects on visual response components like the N2pc (e.g., Luck & Hillyard, 1994, Psychophysiology). That is, we used occipital channels A10/B7, which correspond to PO7 and PO8. In response to the reviewer (and a similar comment from another reviewer), we have clarified the motivation for selecting these specific electrodes. We have also added a new supplemental figure (S4 Fig, pasted under our response to the next comment from Reviewer #1) that shows the topography of the difference wave for distractor-evoked responses during the "good" vs. "bad" alpha phases. This topography confirms that phasic modulation of the broadband response is occurring at the same electrodes as the distractor-evoked response (i.e., the distractor-evoked responses shown in Fig 4C), i.e., at electrodes repeatedly used in previous research on distractor-evoked responses (A10/B7).

From the Results section (lines 141-144): "All analyses of distractor-evoked responses used occipital electrodes A10/B7 (corresponding to PO7/PO8), which were selected a priori based on past work on distractor-evoked visual responses [42–44], as well as past work on other attention-related effects associated with visual responses, such as the N2pc component [45]."

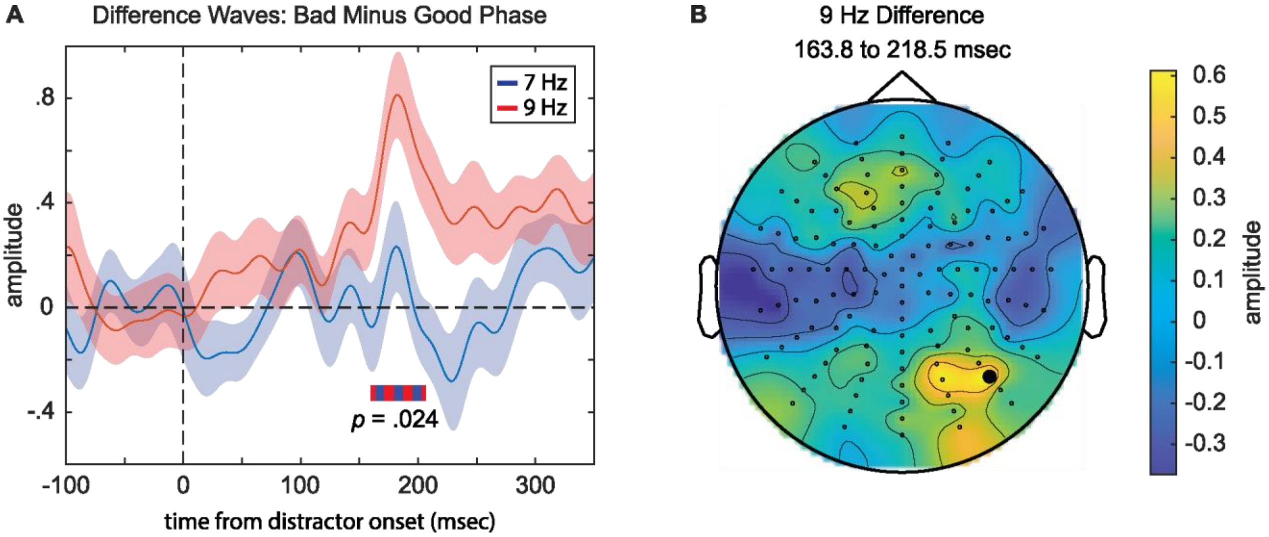

***S4 Fig. Comparison between phasic modulation of distractor-evoked responses at 7 Hz and 9 Hz. A**, Difference waves calculated from distractor-evoked broadband (1–55 Hz) responses as a function of pre-stimulus phase ('good' vs. 'bad') for 7 Hz (blue line) and 9 Hz (red line). Error bars indicate ±1 SEM. Horizontal blue and red bar indicates the time window of significant differences between conditions (159.9 to 206.8 msec). **B**, Topography of the difference wave for the 9 Hz effect averaged over the significant time window (163.8 to 218.5 msec). This plot is configured as if all stimuli appeared on the left side of the visual field (i.e., channels were flipped for trials with stimulus on the right). The electrode used for ERP analyses—A10 or B7 (corresponding to PO7 and PO8), depending on whether the distractor was presented to the left or right side of the visual field—is depicted with a bold dot.*

2. **Reviewer #4**: Temporal definition of pre-stimulus phase

The manuscript reports phase-behavior relationships derived from Morlet wavelet analysis but does not clearly define the temporal window from which phase was extracted. Figure 6 shows results labeled as "pre-stimulus," yet the exact time point or interval used for phase estimation is not specified. It should be clarified whether phase was taken from the final wavelet sample immediately preceding stimulus onset or averaged across a pre-defined period such as −200 to 0 ms. Because wavelets introduce frequency-dependent temporal smearing, the effective center of the extracted phase can differ across frequencies, which is critical for interpreting whether these effects reflect truly anticipatory neural states or early sensory processing. The authors should explicitly state the time-locking reference, the exact latency of phase extraction relative to stimulus onset, and ideally illustrate the sampling window on a time-frequency plot.

We agree with the reviewer that this is an important detail. All of our pre-stimulus wavelets were aligned such that they did not overlap with either the cue-evoked responses or the stimulus-evoked responses. These details can be found in the methods section in the subsection titled, "Lateralized phase-behavior analyses".

From the Methods section (lines 577-583): "Phase was measured on each trial by taking the angle of the complex output produced by convolving the signal with a single frequency-specific Morlet wavelet (3-55 Hz), with a varying number of cycles (2 cycles for 3-8 Hz, increasing logarithmically from 2 to 5 cycles for 9-55 Hz). Subsequent analyses focused on frequencies from 3-25 Hz. We only included trials with a delay period of at least 1000 msec to avoid capturing cue-evoked activity. Wavelets were fit for each frequency at a time point that positioned the last point of the wavelet immediately prior to stimulus onset, to avoid capturing stimulus-evoked activity."

3. **Reviewer #4**: Quantification of phase-behavior coupling
The authors quantify phase-behavior relationships by fitting a one-cycle sine wave to behavioral modulation functions and using the amplitude of this fit as the coupling index. This method assumes that behavioral modulation follows a perfectly sinusoidal pattern, and it may be sensitive to noise or uneven phase-bin sampling. The rationale for choosing this metric over more robust and distribution-free approaches such as circular-linear correlation, phase opposition sum, or ITPC should be explained.

We agree with the review that this analysis specifically tests whether behavioral modulation by pre-stimulus phase follows a one-cycle sinusoidal pattern, with a predicted separation of ~180 degrees between the phases associated with the best and worse behavioral performance. This is a robust approach that has been used in previously published EEG and intracranial NHP studies (e.g., Fiebelkorn et al., 2013; Fiebelkorn et al., 2018; Abdalaziz et al., 2023). In comparison, circular-linear correlations may not be appropriate for non-continuous performance measures like those used here (HR, FAR, and d'). Other measures like the phase opposition sum suffer from reduced power under conditions like those of the present experiment (Van Rullen, 2016, Front. Neurosci.), such as relatively low trial counts (<200) and unbalanced trial counts between conditions (correct and incorrect trials). In response to the reviewer, we have included additional justification in the methods section.

From the Methods section (lines 599-602): "While there are alternative measures of phase-behavior coupling [85], these approaches can suffer from reduced power when trial counts are relatively low (< 200) and unbalanced across conditions (e.g., correct and incorrect trials)."

4. **Reviewer #4** In addition, it should be demonstrated that permutation-based baselines or confidence intervals were applied to ensure that the observed amplitudes exceed what could occur by chance due to random noise. Providing supplementary analyses based on correlation or cluster-level validation would substantially increase confidence in the reported phase-behavior coupling.

Based on this valuable comment (and similar comments from other reviewers), we realized we left out crucial details of the cluster-based permutations tests we used for statistically evaluating the phase-behavior analyses. These are now included in the methods section.

From the Methods section (lines 610-628): "We used cluster-based permutation tests to determine the statistical significance of observed phase-behavior relationships, while correcting for multiple comparisons [86]. First, we used permutation tests to identify frequency x channel pairs with significant phase-behavior relationships. This was done by comparing each observed phase-behavior value (i.e., one-cycle sine fit)—averaged across subjects—with null values obtained by randomly shuffling phases across trials. After shuffling, we recalculated phase-behavior values for each subject and then averaged across subjects. These steps were repeated 1000 times to generate a null distribution. Phase-behavior relationships were considered significant for frequency x channel pairs that exceeded 95% of the null values. Next, we calculated the sum of phase-behavior values in clusters of significant frequency x channel pairs that were neighbors in either frequency or space (i.e., we calculated cluster sums). We then calculated the maximum sum of phase-behavior values in clusters obtained from the null datasets (i.e., the datasets obtained from randomly shuffling phases across trials). For each of the 1000 sets of null phase-behavior functions, we identified frequency x channel pairs with "significant" phase-behavior relationships. As described above for the observed data, null phase-behavior relationships that exceeded 95% of the remaining 999 null values for that frequency x channel pair were considered significant. Null cluster sums were then calculated across frequency and space, and the maximum cluster sum was kept (i.e., we kept the largest cluster sum from each iteration). This was repeated for the remaining 999 null datasets to generate a distribution of maximum cluster sums. Observed cluster sums that exceeded 95% of these null cluster sums were considered statistically significant."

5. **Reviewer #4**: Statistical support for the claim of "distinct oscillatory mechanisms"
The manuscript's central conclusion—that "distinct oscillatory mechanisms phasically modulate the influence of distractors on task performance"—is conceptually appealing but not statistically demonstrated. Theta (around 7 Hz) and alpha (around 9-10 Hz) effects were tested separately, yet there is no direct comparison showing that the two frequency bands differ significantly in spatial topography, magnitude, or behavioral correlation. Without a formal interaction or cross-frequency contrast, the assertion that the mechanisms are "distinct" remains interpretative rather than inferential. A direct comparison between theta- and alpha-band phase-behavior effects, for example via cluster-based permutation testing across frequencies, or a cross-frequency correlation analysis demonstrating independence between the two, would substantiate this key claim. Otherwise, the conclusion should be stated more cautiously as indicating "frequency-specific correlates" rather than distinct mechanisms.

Following the reviewer's suggestion (and similar comments from other reviewers), we now use a cluster-based approach to test for differences in phase-behavior relationships across the distractor and no-distractor conditions, separately for hit rates, false alarm rates, and d'.

From the methods section (lines 629-641): "We also adapted this cluster-based permutation approach to test for differences in the spectral and topographic characteristics of phase-behavior effects observed in different conditions (distractor vs. no distractor). We calculated observed differences by subtracting the strength of the phase-behavior relationships (i.e., one-cycle sine fits) for the no-distractor condition from the corresponding values for the distractor condition. We then obtained null distributions by (i) randomly shuffling trials across the conditions (i.e., across the no-distractor and distractor conditions) for each subject, (ii) recalculating phase-behavior relationships for the shuffled conditions, (iii) averaging phase-behavior relationships across subjects, and (iv) recalculating the between-condition differences.

Frequency x channel pairs with an observed absolute value (i.e., after subtracting strength of phase-behavior relationships for the no-distractor condition from strength of phase-behavior relationships for the distractor condition) greater than 95% of the absolute values of corresponding null pairs were considered significantly different. The calculation of cluster sums and the determination of statistically significant clusters proceeded as described above (i.e., for the analyses of within-condition phase-behavior relationships)."

The results of these additional analyses are now included in the results section and Fig 6.

From the Results section (lines 260-273): "To further test how phase-behavior relationships differed between trials with and without distractors, we used (i) cluster-based permutation tests to compare phase-behavior relationships across trials with and without a distractor and (ii) global dissimilarity measures (DISS) to compare the phase-behavior topographies observed across these same conditions [50]. Cluster-based permutation tests revealed that while central theta effects (d') were apparent for both conditions, these effects were stronger for trials without distractors (Fig 6B-C). Trials with a distractor, in contrast, exhibited significantly greater coupling between alpha phase and all behavioral measures (HR, FAR, and d') at frontocentral electrodes and at posterior electrodes that were contralateral to the distractor (Fig 6B-C). Statistical comparisons of the topographies for phase-d' effects (distractor vs. no-distractor trials), from 6–10 Hz (Fig 7; α = .01), revealed no differences at 6 Hz (DISS = 1.178; p = .393) and 7 Hz (DISS = 1.159; p = .398) and significant differences at 8 Hz (DISS = 1.497; p = .008) and 9 Hz (DISS = 1.485; p = .009). The test for 10 Hz (DISS = 1.469; p = .015) did not survive the correction for multiple comparisons. Combined, these analyses reveal (i) theta-dependent effects on trials with and without a distractor and (ii) alpha-dependent effects that only occurred on trials with a distractor."

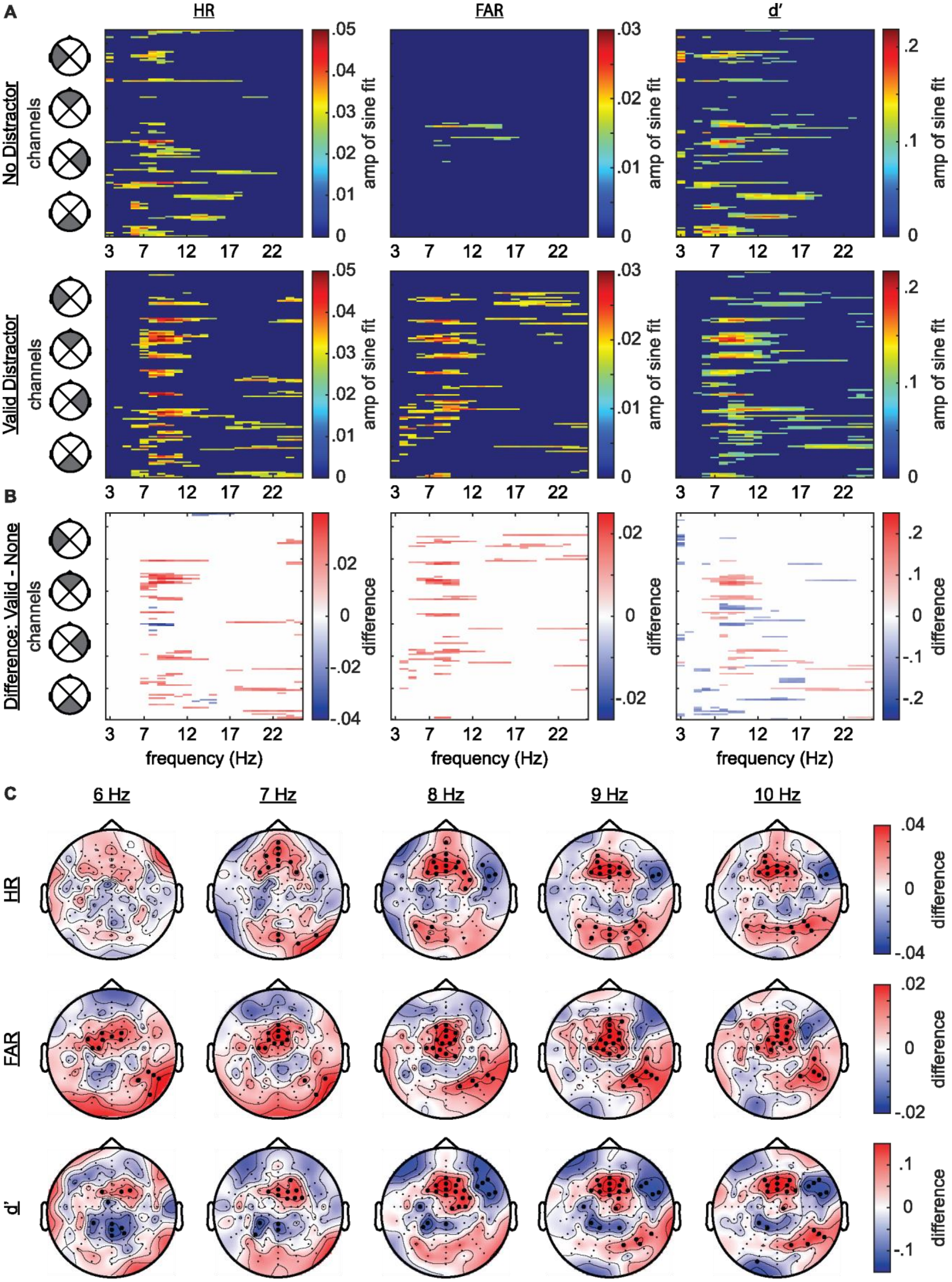
A
HR
FAR
d′
No Distractor
channels
amp of sine fit
Valid Distractor
B
Difference: Valid - None
difference
frequency (Hz)
C
6 Hz
7 Hz
8 Hz
9 Hz
10 Hz

Fig 6. ***Phase-behavior relationships. A****, Plots depicting the strength of statistically significant (p < .05) phase-behavior relationships as a function of frequency (3–25 Hz) across all 128 channels. Statistically insignificant results were set to zero. The first column depicts phase-HR relationships, the second depicts phase-FAR relationships, and the third depicts phase-d' relationships. The top row shows effects for trials with no distractor and the bottom row shows these effects for trials with a valid distractor.* ***B****, Plots depicting differences in phase-behavior coupling between conditions (valid distractor minus no distractor). Statistically insignificant results were set to zero.* ***C,*** *Topographies depicting the differences plotted in* ***B*** *for frequencies from 6-10 Hz (columns). The top row depicts phase-HR differences, the middle row depicts phase-FAR differences, and the bottom row depicts phase-d' differences. Channels with a significant effect at a given frequency are indicated by bold dots. The topographies are configured as if all stimuli appeared on the left side of the visual field (i.e., channels were flipped for trials with stimulus on the right).*

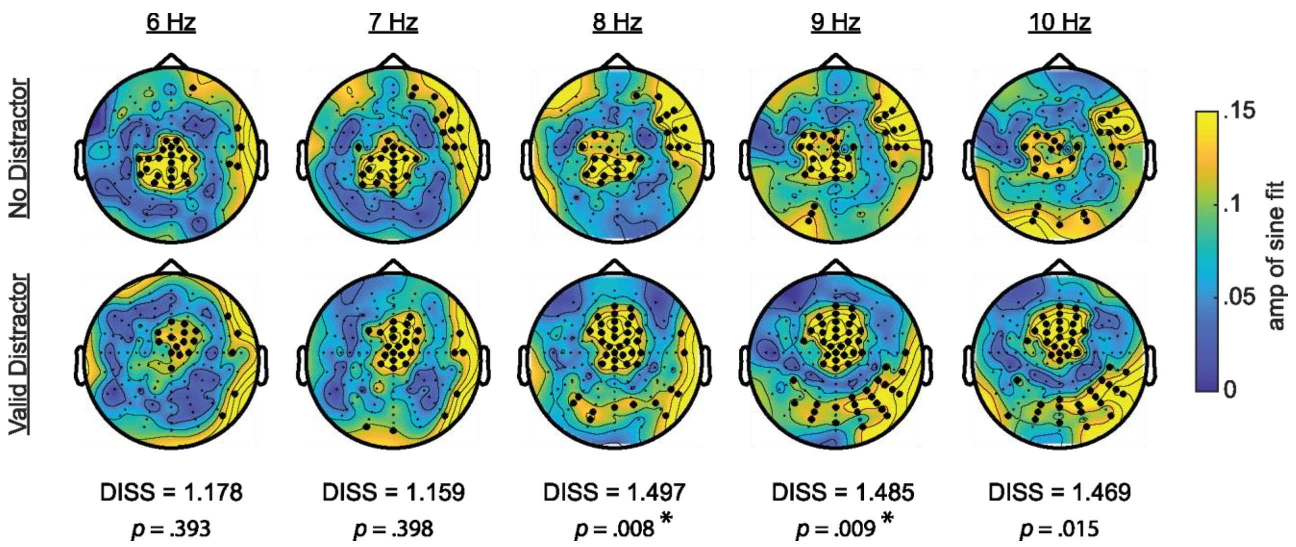

Fig 7. Topographic analyses. *Scalp topographies show the strength of phase-d' relationships for frequencies from 6–10 Hz (columns). The top row shows effects for trials with no distractor and the bottom row shows effects for trials with a valid distractor. Channels with a significant effect at a given frequency are indicated by bold dots. Global dissimilarity measures (DISS) and p-values for tests comparing topographies across the no-distractor and valid-distractor conditions are listed below the respective frequency, with significant values after correcting for multiple comparisons (p < .05) indicated with an asterisk. The topographies are configured as if all stimuli appeared on the left side of the visual field (i.e., channels were flipped for trials with stimulus on the right).*

Further functional differences between 'theta effects' and 'alpha effects' emerge when evaluating whether there is phasic modulation of distractor-evoked visual responses. Whereas theta phases associated with better and worse behavioral performance showed no effect on the visual responses to salient distractors (Fig 8D), the alpha phase associated with worse behavioral performance was associated with stronger distractor responses, relative to the alpha phase associated with better behavioral performance (Fig 9D).

6. **Reviewer #4**: Lack of explicit statistical test for phase-behavior relationships

Although the manuscript refers to significant phase-behavior relationships in several figures, the precise statistical test producing these results is not described in sufficient detail. It remains unclear what statistic was used to evaluate significance—whether it was based on the sine-fit amplitude across participants, circular-linear correlations between phase and behavior, or differences between phase bins relative to a null model. The construction of the null hypothesis also needs clarification: were phases shuffled within participants, or were behavioral labels permuted to create surrogate datasets? Furthermore, the manuscript does not specify how multiple comparisons were handled given the large search space across 128 electrodes, a 3-25 Hz frequency range, and three behavioral measures (HR, FAR, and D'). The absence of reported effect sizes or group-level variability further limits assessment of robustness. To substantiate the claim of significant phase-behavior coupling, the authors should clearly state the test statistic, the structure of the null distribution, and the correction method applied across frequencies and channels; otherwise, these results should be treated as descriptive rather than inferential.

We apologize to all of the reviewers that we failed to include a section describing our cluster-based approach to statistics, which we used for all of the phase-behavior analyses. To summarize, the sine-fit amplitude was compared against null values obtained by first shuffling phase across trials (at the subject level) and then recalculating phase-behavior relationships. Cluster-based permutation tests were used to account for multiple comparisons. As indicated in a previous response to Reviewer #4, we have added the following text to the manuscript:

From the Methods section (lines 610-628): "We used cluster-based permutation tests to determine the statistical significance of observed phase-behavior relationships, while correcting for multiple comparisons [86]. First, we used permutation tests to identify frequency x channel pairs with significant phase-behavior relationships. This was done by comparing each observed phase-behavior value (i.e., one-cycle sine fit)—averaged across subjects—with null values obtained by randomly shuffling phases across trials. After shuffling, we recalculated phase-behavior values for each subject and then averaged across subjects. These steps were repeated 1000 times to generate a null distribution. Phase-behavior relationships were considered significant for frequency x channel pairs that exceeded 95% of the null values. Next, we calculated the sum of phase-behavior values in clusters of significant frequency x channel pairs that were neighbors in either frequency or space (i.e., we calculated cluster sums). We then calculated the maximum sum of phase-behavior values in clusters obtained from the null datasets (i.e., the datasets obtained from randomly shuffling phases across trials). For each of the 1000 sets of null phase-behavior functions, we identified frequency x channel pairs with "significant" phase-behavior relationships. As described above for the observed data, null phase-behavior relationships that exceeded 95% of the remaining 999 null values for that frequency x channel pair were considered significant. Null cluster sums were then calculated across frequency and space, and the maximum cluster sum was kept (i.e., we kept the largest cluster sum from each iteration). This was repeated for the remaining 999 null datasets to generate a distribution of maximum cluster sums. Observed cluster sums that exceeded 95% of these null cluster sums were considered statistically significant."

7. **Reviewer #4**: Asymmetry between phase and power analyses
Phase-behavior effects were observed for both theta and alpha frequencies, yet the power analysis was performed only for the alpha band. This asymmetry weakens the argument for frequency-specific mechanisms. If both frequencies were behaviorally relevant in the phase domain, their corresponding power components should also be examined to determine whether amplitude dynamics show similar or different relationships to behavior. By limiting the power analysis to alpha, the authors risk post hoc selection consistent with existing expectations about alpha suppression, rather than an unbiased

evaluation across frequencies. Without testing theta power, it is impossible to know whether the null alpha-power result is specific to that band or reflects a general absence of power-behavior coupling. The authors should either justify theoretically why only alpha power was analyzed or include parallel theta-band power analyses using the same framework. This would make the argument for distinct frequency-specific mechanisms considerably more convincing.

The purpose of this study was to investigate changes in behavioral performance as a function of oscillatory phase. That is, we set out to specifically test a hypothesis that distractibility fluctuates as a function of oscillatory phase—a prediction of the Rhythmic Theory of Attention. While testing this central hypothesis, we found alpha-phasic modulation of behavioral performance that specifically occurred contralateral to the distractor location (i.e., alpha-phasic modulation was lateralized). In comparison, there was no evidence that theta-phasic modulation was dependent on the hemifield of stimulation. There is an ongoing debate in the literature regarding the role of alpha-band activity in sensory processing, specifically with respect to distractor suppression. A key point in this debate revolves around whether alpha-associated suppression functions through power modulation and/or phasic modulation. Given typical reports of lateralized alpha power associated with the deployment of spatial attention and the observed lateralization of the present alpha-phasic modulation, it became important to test whether lateralized alpha power was also linked to behavioral performance. As there was no evidence of lateralized theta-phasic modulation (and no ongoing debate regarding the role of lateralized theta-band activity), we did not test whether lateralized theta power was linked to behavioral performance. In response to the reviewer, we have clarified our motivation for specifically testing whether there was a relationship between alpha power and behavioral performance.

From the Results section (lines 292-313): "While alpha-band activity has been repeatedly linked to the suppression of sensory processing, evidence for a relationship between alpha-band activity and distractor suppression has been mixed [40]. In the previous analysis, the results demonstrated a significant relationship between pre-distractor alpha phase and subsequent behavioral and neurophysiological responses (Figs 6 and 9). In addition to frontal electrodes, these effects occurred at electrodes contralateral to the presentation of distractors. This is consistent with lateralized alpha-band activity during the deployment of spatial attention [33]. Given ongoing debates regarding whether alpha-associated suppression functions through phasic modulation and/or power modulation, we tested whether pre-stimulus alpha power was also linked to behavioral performance on trials with distractors. We specifically tested whether alpha-power lateralization (i.e., alpha power at electrodes contralateral to the distractor cue relative to alpha power at electrodes ipsilateral to the distractor cue) was linked to behavioral performance. Power spectra for five subjects did not display obvious alpha-band peaks. These subjects were therefore excluded from the present analyses (n = 27). The mean peak alpha frequency (across subjects) was 10.95 Hz (Fig 10A). The topography for alpha-power lateralization confirmed greater power over occipital electrodes that were contralateral to the distractor cue (Fig 10B), consistent with previous findings [51]; however, alpha-power lateralization at the occipital electrodes with the greatest alpha lateralization (A10/B7) was not associated with behavioral performance on trials with distractors [HR: $t_{(26)} = 0.164$, $p = .871$, $d = .032$; FAR: $t_{(26)} = 0.892$, $p = .38$, $d = .135$; d': $t_{(26)} = 0.056$, $p = .956$, $d = .047$] (Fig 10C-E). These results indicate that alpha-power lateralization in the present experiment—unlike alpha phase (see the previous analysis)—was not associated with suppression of distractor processing. As there was no evidence of lateralized phasic modulation within the theta-frequency band, we did not conduct similar tests based on lateralized theta power."
