# Supplementary material for: Frequency-specific attentional mechanisms phasically modulate the influence of distractors on task performance
Source: PLoS Biol. 2026 Feb 23;24(2):e3003664. doi: 10.1371/journal.pbio.3003664 (PMC12928401; doi:10.1371/journal.pbio.3003664)
Supplement: Attachment — Submitted filename: Response_to_Reviewers_auresp_2.pdf [file pbio.3003664.s010.pdf]

Editorial Requests:

* We would like to suggest a different title to improve its accessibility for our broad audience:
Frequency-specific attentional mechanisms phasically modulate the influence of distractors on task performance

We agree that this title represents our findings well and should be appealing to the broad readership of PLOS Biology. We have included this change in the manuscript (lines 4-5).

* Please add the links to the funding agencies in the Financial Disclosure statement in the manuscript details.

This information has been included in the revised submission.

* Please include the approval/license number of the ethical approval for the experiments.

This information has been included in the revised submission.

* Please include information in the Methods section whether the study has been conducted according to the principles expressed in the Declaration of Helsinki.

This information is now specified in the manuscript (line 467).

* Please specify whether the participants provided written or oral consent.

Participants provided written consent. This information is now included in the manuscript (line 467).

* DATA POLICY:

1) Supplementary files (e.g., excel). Please ensure that all data files are uploaded as 'Supporting Information' and are invariably referred to (in the manuscript, figure legends, and the Description field when uploading your files) using the following format verbatim: S1 Data, S2 Data, etc. Multiple panels of a single or even several figures can be included as multiple sheets in one excel file that is saved using exactly the following convention: S1_Data.xlsx (using an underscore).

We have created 3 excel files with the individual scores used for behavioral analyses (see below).

2) Deposition in a publicly available repository. Please also provide the accession code or a reviewer link so that we may view your data before publication.

Preprocessed data have been uploaded to the Open Science Framework (doi: 10.17605/OSF.IO/V24GU); however, due to limitations in the amount of data that could be uploaded, we could not upload the raw data files. Interested parties will be welcome to contact us for the raw data.

Regardless of the method selected, please ensure that you provide the individual numerical values that underlie the summary data displayed in the following figure panels as they are essential for readers to assess your analysis and to reproduce it: 2ABC, 3ABCDEF and 10CDE.

We have created excel files with the individual scores used to create the figures referenced. We have included references to these supplemental data files in the results section of the manuscript and in the respective figure legends. S1 Data includes data for Fig 2A-C (lines 92-93 and 109-110). S2 Data includes data for Fig 3A-F (lines 118-119, 124, and 137). S3 Data includes data for Fig 10C-E (lines 312-313 and 327).

Please also ensure that figure legends in your manuscript include information on where the underlying data can be found, and ensure your supplemental data file/s has a legend.

Figure legends now include references to the excel files that contain relevant supplementary data.

Please ensure that your Data Statement in the submission system accurately describes where your data can be found.

We have included the DOI for deposited code and data in the Open Science Framework (doi = 10.17605/OSF.IO/V24GU).

* CODE POLICY

Per journal policy, if you have generated any custom code during the course of this investigation, please make it available without restrictions. Please ensure that the code is sufficiently well documented and reusable, and that your Data Statement in the Editorial Manager submission system accurately describes where your code can be found.

We have uploaded custom matlab codes used for our analyses to the Open Science Framework alongside the preprocessed data (doi = 10.17605/OSF.IO/V24GU). This includes the scripts used for preprocessing the raw EEG data and scripts for all analyses included in the manuscript.

Reviewer Comments:

Reviewer #1 (signed as Biao Han): The authors have addressed the main methodological concerns raised in my initial review. The revised manuscript now clarifies several critical analysis choices, including the a priori rationale for ERP electrode selection, the temporal definition of pre-stimulus phase estimation, and the statistical framework used to evaluate phase-behavior relationships. The inclusion of cluster-based permutation procedures and additional control analyses improves the robustness and transparency of the reported effects.

The authors also provide formal statistical comparisons supporting frequency-specific differences between theta- and alpha-band effects, which strengthens the interpretability of the central claims. While some analyses could in principle be extended further, the current revisions are sufficient to support the conclusions as stated.

We would like to thank this reviewer (Biao Han) once again for their excellent suggestions which we believe have substantially strengthened the presentation of our findings.

Reviewer #2: The authors have done a good job of addressing all my comments, except for my first point. Apparently, I did not formulate it clearly enough. My point was that it would be desirable for the authors to provide an analysis of behavioral oscillations, such as in Figure 2 in Fiebelkorn et al. 2018 and Figure 1 in Helfrich et al, 2018. Such time-resolved behavioral time courses would provide a solid functional basis for the fact that distractor susceptibility fluctuates over time.

We would first like to thank this reviewer once again for their very constructive comments. We would also like to apologize for our misinterpretation of this reviewer's previous comment about the inclusion of behavioral oscillations. While we and others have shown evidence of rhythmic fluctuations in behavioral performance (e.g., Landau and Fries, Current Biology, 2012; Fiebelkorn et al., Current Biology, 2013), this approach typically requires very high trial counts in order to reconstruct behavioral performance as a function of different cue-target delays. Behavioral outcomes from each trial (e.g., hits and misses) need to be sorted into different time bins—based on the cue-target delay—and each time bin needs to have enough trials to reliably calculate the behavioral measure (e.g., hit rate). For the present study, we would need to separately calculate behavioral oscillations occurring during trials with and without distractors, with our combined electrophysiological and behavioral results suggesting that effects might occur at distinct frequencies for these conditions (i.e., ~7 Hz on trials without a distractor, additional ~9-10 Hz oscillations on trials with a distractor). Given that we planned to correlate behavioral performance with electrophysiological measures (e.g., oscillatory phase), which requires fewer trials, we did not collect a sufficient number of trials to test for behavioral oscillations. Specifically, we would only have an average of ~15 trials per time bin for each subject (using 50-ms time bins, as we have previously done). While we certainly agree with the reviewer that behavioral oscillations can provide a strong demonstration of the behavioral relevance of oscillatory neural activity (i.e., by visualizing oscillations in the behavioral data itself), there are multiple reasons why we believe it is better to correlate behavioral performance with recorded neural activity. I have previously written an opinion piece on this specific issue (Fiebelkorn, 2022, "There is more evidence of rhythmic attention than can be found in behavioral studies: Perspective on Brookshire," Journal of Cognitive Neuroscience). For example, correlating behavioral performance with electrophysiological measures (e.g., oscillatory phase) reveals important information about how different brain regions contribute under different experimental conditions (even when the functionally relevant frequencies are similar across experimental conditions)—as we have observed in the present study. Behavioral oscillations, in comparison, provide no information about underlying neural sources. We again thank the reviewer for their thoughtful comment, but we cannot calculate behavioral oscillations, given the trial counts, and we strongly believe that our combined electrophysiological and behavioral results (e.g., the coupling of pre-stimulus phase with hit rates, false alarms, and d') already provide compelling evidence for rhythmic distractor susceptibility.

Reviewer #3 had no further comments.

We would like to thank this reviewer for their helpful comments in the initial review.
